# Silane modification of TiO_2_ nanoparticles and usage in acrylic film for effective photocatalytic degradation of methylene blue under visible light

**DOI:** 10.1038/s41598-023-34463-7

**Published:** 2023-05-06

**Authors:** Maryam Rostami Ataabadi, Masoud Jamshidi

**Affiliations:** Constructional Polymers and Composites Research Lab., School of Chemical, Petroleum and Gas Engineering, Iran University of Science and Technology (IUST), Tehran, Iran

**Keywords:** Environmental sciences, Materials science, Nanoscience and technology

## Abstract

To fabricate a photocatalytic acrylic paint, TiO_2_ nanoparticles were surface modified by a bi-functional amino silane (i.e. bis-3-(aminopropyltriethoxysilane)) at different concentrations and applied at 1, 3 and 5 wt% to an acrylic latex. It was found that the surface modification of nano TiO_2_ enhanced its specific surface area about 42%. The tensile properties of the pristine and nanocomposite acrylic films were assessed. The photocatalytic degradation of aqueous solution and stain of methylene blue (MB) were evaluated (under solar, visible, and UV illuminations) by nanoparticles and nanocomposites, respectively. Results showed that incorporating 3 wt% of the pure and modified nano TiO_2_ to arylic film caused 62 and 144% increment in the tensile strength. The modified nanoparticles showed higher MB degradation contents under UV, visible and solar irradiation (82, 70, 48%, respectively). The addition of pure and modified nanoparticles to the acrylic film caused decrement in the water contact angle from 84 to 70 and 46°, respectively. It also caused considerable enhancement in the glass transition temperature (*T*_*g*_) of acrylic film compared to the pristine and pure nanocomposite films (i.e. about 17 and 9 °C, respectively). Furthermore, it was found that the modified nanocomposite caused more color change of MB stain (65%).

## Introduction

In the last century, with the growth of human population and industrialization, concerns for environmental pollutions (e.g. air and water pollutants) have attracted a lot of attentions^1–3^. Various kinds of contaminants are being released into the surface water mainly because of human activities that threaten the ecosystem. A group of these contaminants are dyes that discharged from different industries such as leather, textile and plastics manufacturers into the water resources. Methylene blue (MB) is the most commonly employed material for dying wood, silk, and, cotton which might be responsible for permanent eye injury in animals and humans. Hence, the dye-based contaminants in wastewater should be treated to mitigate its detrimental impacts^4^.

Many efforts have been accomplished for treating these contaminants to find effective methods to control and eliminate them from wastewater^5–7^. Photocatalytic oxidation (PCO) is a relatively novel technique that has been used to eliminate water pollutants in recent years^8^. In this method, a semiconductor catalyst is activated by light to oxidize the pollutant^9^. The typical photocatalysts are sulfides and metal oxides (e.g. TiO_2_, ZnO, ZrO_2_, SnO_2_, WO_3_, CeO_2_, Fe_2_O_3_, Al_2_O_3_, ZnS, and CdS) that TiO_2_ and ZnO are the most popular ones among them^10^.

Titanium dioxide (TiO_2_) is one of the promising photocatalysts that has excellent photocatalytic activity primarily under ultraviolet (UV) light^11–13^. Its exceptional characteristics (e.g. non-toxicity, high oxidation behavior, photo-stability, chemical inertness and good cost-quality balance) made it much popular. By reducing the size of TiO_2_ particle to the nano scale, its photocatalytic activity improves due to the increase in the effective surface area^14^.

The photocatalytic behavior of TiO_2_ has received much more attentions due to its potential in treating different wastes and pollutants using solar energy^15–17^. Anatase crystalline phase of TiO_2_ showed highly effective in preparation of electron–hole pairs. The electrons and holes produce radical and ionic specious in reaction with H_2_O and O_2_ that they could decompose water/air pollutation. However, rutile phase showed stability under lights irradiation. Nowadays, the P-25, as a standard TiO_2_ nanoparticle that consists of both rutile and anatase phases (mainly with the ratio of 30:70 or 20:80) is usually used in photocatalytic applications^18^.

When TiO_2_ is exposed to UV light, it generates hole-electron pairs in which the hole enjoys abundant oxidizing power while the electron possesses strong reducing power. Considering this, the synergistic effect of electron–hole pairs could be a hotspot for removing organic pollutants and reducing heavy metals. However, adsorption of methylene blue (MB) molecules to TiO_2_ surface is low because of several hydroxyl groups placed on the photocatalyst surface^19^. Therefore, finding a solution to enhance photocatalytic activity of TiO_2_ for degradation of aqueous solution of MB is critical.

Although, direct using of photocatalyst nanoparticles for degradation of dyes is more effective but their separation from purified water and also their agglomeration in wastewater are two challenging issues^20^. To solve the first problem, many researches have been performed to immobilize these nanoparticles on different surfaces such as concretes^21,22^, asphalts^23^, rubbers^24^, plastics^25,26^, carbon cloth^27^ and metals^28^. To eliminate agglomeration of hydrophilic nanoparticles in aqueous condition, coupling agents (e.g. silanes) and surface acting agents have been normally used^29–31^.

Paints and coatings are used traditionally for decoration and/or protection of substrates in different industries. They are applied in thin layers on huge surfaces that exposed to air and water. On this basis, some efforts have been performed to use photocatalysts in polymers to prepare photocatalytic thin films for degradation of organic pollutants.

Pazokifard et al.^32^ investigated the surface modification of titanium dioxide nanoparticles with perfluorooctyltriethoxysilane (fluorosilane) under alkaline conditions. According to this research, modified nanoparticles were added to a water-based acrylic coating to prepare a photocatalytic paint. The photocatalytic activity of the films was investigated by evaluating decomposition of Rhodamine B under UVA radiation as a model pollutant on the surface of the paint. It was found that silane grafting on the nanoparticles improved photocatalytic performances of the acrylic films.

Dianatdar et al.^33^ prepared acrylic based pseudo paint to remove benzene from air. They determined the optimal amount of TiO_2_ pigments and nanoparticles to improve mechanical and photocatalytic properties of acrylic films under UV light. Arekhi et al.^34^ added water glass as an inorganic binder (at 5 and 10 wt%) to acrylic latex, in order to improve dispersion of TiO_2_ nanoparticles in the polymer film. They found that addition of 5 wt% of water glass increased the photocatalytic activity and benzene removal of the acrylic nanocomposites under UV light.

Hashemi Monfared et al.^35^ investigated the changes in the mechanical properties of acrylic latex contained PAni/TiO_2_ and nano TiO_2_ after the photocatalytic degradation of benzene under UV light. The results showed that the mechanical properties of samples contained PAni/TiO_2_ decreased due to the weak interactions between the polymer and nanoparticles. Besides, PAni/TiO_2_ containing nanocomposites showed less benzene removal compared to the pure nano TiO_2_ containing sample due to the agglomeration of the nanoparticles. Habibi et al.^36^ prepared TiO_2_ nano fibers using cellulose nanofibers as template. They prepared acrylic nanocomposites films contained TiO_2_ nano fibers, polyaniline or Pani/TiO_2_ to remove organic dye under UV, visible and solar lights irradiation. The results showed that the nanocomposites contained pure polyaniline had acceptable absorption in all wavelengths and the presence of polyaniline in the structure of the nanocomposite caused the absorption of light in higher wavelengths. However, the agglomeration of nanoparticles would be intensified by inclusion of hydrophilic nano photocatalyst in a hydrophobic polymer matrix.

Numerous researchers have been performed to improve dispersion of TiO_2_ nanoparticles in polymeric films. It has been found that it is possible to use organic functional groups to create chemical bonds on the surface of the nanoparticles to eliminate their agglomeration^37–39^. Wanag et al.^18^ used 3-aminopropyltriethoxysilane (APTES) to modify the surface of titanium dioxide. TiO_2_/APTES photocatalysts have been produced using solvothermal method. It was generally observed that surface modification of TiO_2_ by APTES improved its photocatalytic activity. Zhao et al.^38^ modified the surface of TiO_2_ nanoparticles by (IPTMS) and (APTMS) in order to enhance the sustainability of nanoparticles on the fabric's surface. There was a slight decline in the photocatalytic reaction rate constant of the later one with an increase in organosilane content from 0 to 200 wt%. A rapid decrease in the photocatalytic activity of IPTMS modified TiO_2_ was obtained by increment in the silane grafting ratio.

It has been known that silane coupling agents could deposit a thin layer on the surface of some inorganic oxides that affects their photocatalytic performances. TiO_2_ nanoparticles are the most interested photocatalyst by industries. However, there are few investigations on effects of silane grafting on photocatalytic performances of TiO_2_ nanoparticles. Moreover, silane grafting of nano TiO_2_ and its effect on the mechanical and photocatalytic performances of the prepared polymeric nanocomposites has not been investigated, yet.

In this work, Bis-(triethoxysilylpropyl) amine (BTESPA, denoted by *A* in this work) was used as a bifunctional amine based silane coupling agent for surface modification of P-25 nanoparticles for the first time. The silanization was performed to induce steric stabilization to the nanoparticles and eliminate their agglomeration in aqueous environment and acrylic film. To increase silane grafting content, silanization was performed at different silane concentrations (i.e. 1, 3, 5 and 7 times to stoichiometric content). The pure and silanized P25 nanoparticles were characterized using Fourier transform infrared spectroscopy (FTIR), X-ray diffraction (XRD), thermogravimetric analysis (TGA), Brunauer–Emmett–Teller (BET) technique and Field Emission Scanning Electron Microscopy (FE-SEM) with Energy Dispersive X-Ray Spectroscopy (EDS). The pure and silanized nanoparticles were applied at 1, 3 and 5 wt% to acrylic latex as conventional matrix for water-based paints. The prepared nanocomposite films were evaluated for mechanical properties and photocatalytic degradation of methylene blue stain (i.e. under UV, visible and solar lights). The characteristics of the optimized samples were studied by FE-SEM, atomic force microscopy (AFM), dynamic mechanical thermal analysis (DMTA), differential reflectance spectroscopy (DRS) and contact angle test.

## Experimental work

### Materials

Acrylic latex (R-84), as polymeric binder, was supplied from Simab Resin Company (Iran) (45 ± 2% solid content, MFFT of 16 °C and mean particle size of 0.8–1 μm). TiO_2_ nanoparticles (P-25) as photocatalyst with a mean particle size of 21 nm, anatase to rutile ratio of 85:15 and specific surface area of 50 ± 10 m^2^/g was purchased from Evonic Co. (Germany). Bis-(triethoxysilylpropyl)amine (BTESPA) was purchased from Evonic Co. (Germany). Methylene blue (MB) as a model pollutant of wastewater was purchased from Merck Company (Germany). Acrylic homopolymer (D-200) with a molecular weight of 5000 g/mol produced by Simab Resin Co. (Iran) was employed as dispersant of the nanoparticles in acrylic latex.

### Methods

#### Surface modification of TiO_2_ nanoparticles

1 g of TiO_2_ nanoparticles was dispersed in 100 mL ethanol and sonicated for 30 min. the suspension was poured in a glass round-bottom flask equipped with a condenser and refluxed during heating in an oil bath at 75 °C for 4 h. The silane solution in ethanol was added dropwise to the suspension for hydrolysis of silane molecules on the nanoparticles surfaces.

The stoichiometric amount of silane (X) to react with all surface OH groups of the nanoparticles was determined based on the Mrkoci^40^ equation:

1 $$ m_{S} = X\left( {\frac{{6 \times S_{T} \times m_{T} \times M_{S} \times OH_{N} \times 10^{19} }}{{N_{A} }}} \right) $$

where *m*_*S*_ is the mass of silane )g(, *S*_*T*_ is the surface area of TiO_2_, *m*_*T*_ is the mass of TiO_2_ )g( and *M*_*S*_ is the molecular weight of silane. The term *OH*_*N*_ depicts the number of hydroxyl groups per nm^2^ of the nanoparticle surface, N_A_ is Avogadro number and the value of 10^19^ is a dimensional conversion factor. Silanization of nanoparticles was performed at silane concentrations of 1, 3, 5 and 7×.

The surface modified nanoparticles were separated from solvent by centrifuging at a rate of 3500 rpm for 15 min. Then, the separated particles were washed with ethanol and acetone three times to eliminate excessive unreacted silane molecules. To complete silane condensation step, the modified nanoparticles were finally dried at ambient temperature for a day and then in an oven at 80 °C for 12 h.

#### Fabrication of photocatalytic nanocomposite films

To prepare the nanocomposite samples, dispersant and TiO_2_ nanoparticles were dispersed in deionized water via an ultrasonic homogenizer (Bandelin 3200, with VS 70 T probe, Germany). Then, NaOH was added to the solution to adjust the pH value to 10. After 30 min of sonication (at 30 W), the suspension was added to the acrylic latex at different concentrations (1, 3 and 5 wt%) (see Table 1) and stirred for an hour. The samples were stirred at 900 rpm for 60 min. A pure acrylic film was prepared as control sample for comparison.

**Table 1:** Formulation of the prepared photocatalytic nanocomposite films.

Sample code	Acrylic latex (g)	Dispersant (g)	Photocatalyst (g)	
			Pure	Silanized
AC	98.6	0.4	0	0
AC-NT1	98.6	0.4	1	0
AC-NT3	98.6	0.4	3	0
AC-NT5	98.6	0.4	5	0
AC-NT1-A(5X)	98.6	0.4	0	1
AC-NT3-A(5X)	98.6	0.4	0	3
AC-NT5-A(5X)	98.6	0.4	0	5

The nanocomposite films were applied on glass plates with a film applicator at wet film thickness of 400 μm (i.e. final dry film thickness of about 200 μm (± 3 μm)). They were cured at ambient temperature in dark conditions for a whole day and then separated from the plate. For tensile test, ten specimens (75 mm in length and 150 mm in width) were prepared for each sample based on the ASTM standard method^41^.

### Tests and characterizations

A sealed glass box with dimensions of 50 cm × 40 cm × 30 cm was used as batch photocatalytic reactor in this study^31^. The photo reactor was equipped to five 8 W visible lamps (i.e. totally 40 W) and three 8W UV-C lamps (i.e. totally 24 W). To evaluate photocatalytic degradation of methylene blue, a stain of aqueous solution of methylene blue (i.e. at concentration of 10 mg/lit) was dripped on the nanocomposite films. The films were placed in the photo reactor under desired light for 24 h.

To determine effect of silane grafting on the photocatalytic performances of the nanoparticles, the pure and modified nanoparticles (0.125 g) were added to methylene blue aqueous solutions (i.e. at concentration of 10 mg/lit). The samples were placed under UV, visible and solar lights and evaluated for color changes by UV–Vis spectrophotometry (Photonix, Ar 2015) at λ_max_ = 663 nm at different exposure times^34^. The photocatalytic activity under solar light irradiation was performed under natural sunlight from 8 a.m. to 13 p.m. (i.e. totally 5 h).

The degradation of MB could occur by both photolysis and photo-degradation. Hence, the photo-degradation of MB was determined under lights illuminations in absence of photocatalyst. The aqueous solutions containing MB and dispersed photocatalysts were fixed in dark condition for 1 h before turning the lights in order to achieve the MB adsorption content by the nanoparticles.

The efficiency of photo-degradation, i.e., *E%*, of each photocatalyst was determined by the following equation^42^:

2 $$ E\left( \% \right) = \frac{{A_{0} - A_{t} }}{{A_{0} }} \times 100 $$

where *A*_*0*_ is the light absorbance of MB solution when turning off the light, and *A*_*t*_ is its light absorbance at time *t* when turning on the light.

The color changes in the MB stains placed on the nanocomposite films was measured by X-Rite device (Taiwan). The total color change (ΔE*) was calculated by Eq. (3) as following:

3 $$ \Delta E^{*} = \sqrt {\left( {\left( {\Delta a^{*} } \right)^{2} + \left( {\Delta b^{*} } \right)^{2} + \left( {\Delta L^{*} } \right)^{2} } \right)} $$

where Δa*, Δb*, and ΔL* are the changes in a*, b*, and L* baseline values, respectively^43^.

The tensile properties of the prepared films were evaluated based on ASTM D638^44^. The tensile strength (*TS*), elongation at break (*EB*) and Young modulus (*S*) of all the samples were determined. The test was performed using SANTAM STM150 universal machine at tension rate of 5 mm/min. the obtained load (N)-deflection (mm) curves were used to calculate tensile strength, toughness and modulus contents.

The morphology of the pure and silanized nanoparticles were studied using FE-SEM analysis (TESCAN MIRA VIII device equipped with EDS, Czech Republic). The nanocomposite films were fractured in liquid nitrogen (N_2_) and the cross-sections were also characterized using FE-SEM analysis. The thermomechanical properties (DMTA) of the nanocomposites were measured by a Triton Tritec 2000 DMTA apparatus (UK). Thermogravimetric properties (TGA/DTA) of the acrylic films were investigated via a BAHR STA 504 (Iran) apparatus in the temperature range of 25–600 °C with a fixed heating rate of 10 °C/min in air condition. The chemical structures and functional groups were characterized using Fourier transform infrared spectroscopy (FTIR) by a Perkin Elmer PX1 spectrometer (USA). The X-ray diffraction (XRD) patterns were obtained by PHILIPS PW-1730 X-ray diffractometer (USA) with CuKα radiation (λ = 1.5418 Å) in the 2θ range of 10–80°. The optical characterizations were recorded using a UV–Vis diffuse reflection spectrophotometer (UV–Vis DRS) by a Shimadzu UV2550 spectrometer (Japan). The water contact angle (WCA) of the films were measured using a drop shape analyzer, Data Physics instrument (DSA30E, Kruss Co. Ltd., Hamburg, Germany).

## Results and discussions

### Characterization of nanoparticles

**Figure 1:**
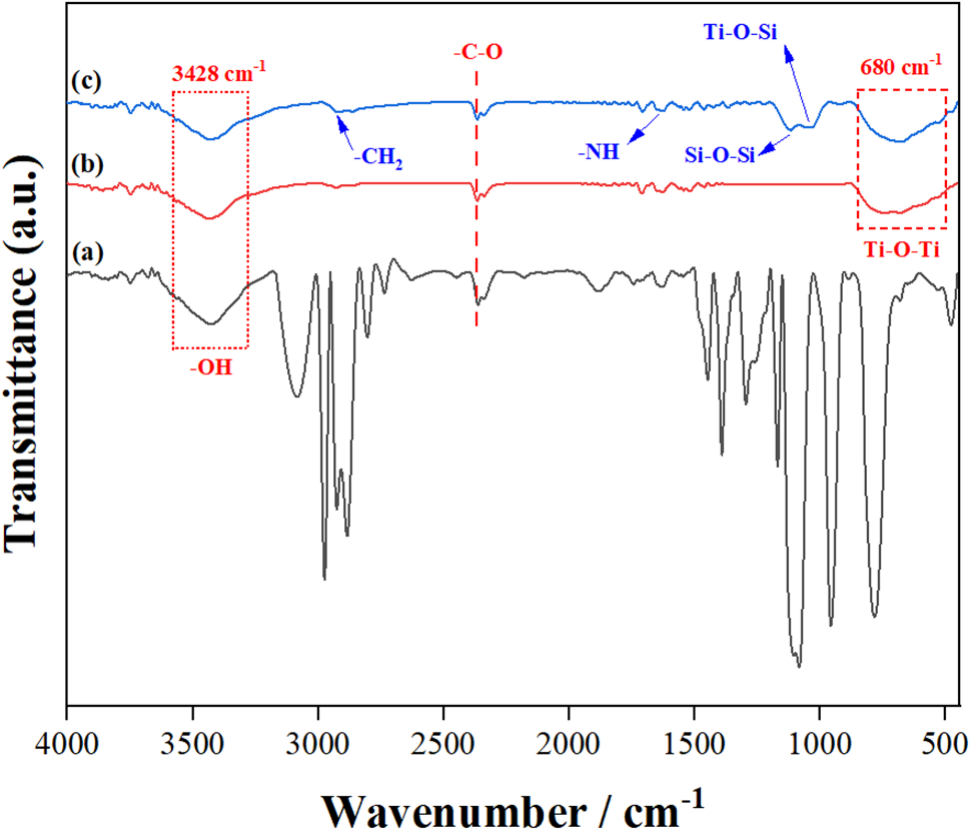
FTIR spectra of (**a**) Silane (i.e. BTESPA), (**b**) pure TiO_2_ (i.e. NT), and (**c**) modified TiO_2_ nanoparticles (i.e. NT-A(5X)).

Figure 1 depicts the FTIR spectra of BTESPA, pure and modified TiO_2_ nanoparticles. The broad peak at wavelength of 3470 cm^−1^ was corresponded to the existence of some water in the silane structure due to this fact that it has hydrophilic nature and absorbs humidity (see Fig. 1a). The stretching vibration peak of NH group usually was seen close to this zone but here it has been overlapped by OH related broad peak. The peak that was seen at wavelength of 1630 cm^−1^ was related to bending vibration of OH group of adsorbed water. The peak that was placed at about 3200 cm^−1^ was related to C–H stretching vibration of (in ethoxy groups) in silane structure. The observed peaks between 2800 and 3050 cm^−1^ were related to stretching vibration of CH and CH_2_ groups (in propyl and ethoxy groups) of the silane. The peak at 1116 cm^−1^ shows presence of Si–O–Et group in the chemical structure of silane molecules.

Figure 1b shows FTIR spectrum of the pure TiO_2_ nanoparticle. It is obvious that there is a broad band at 3450 cm^−1^ that should be related to covalently bonded OH groups to the surface of nanoparticles. This peak was decreased in the silanized nanoparticles somehow that confirmed chemical reaction between hydrolyzed silane molecules (see Fig. 1c). Furthermore, the peaks obtained at 1000–1200 cm^−1^ was attributed to Si–O–C vibration that has been created on the nanoparticles surface by hydrolysis of silane molecules. Besides, the peaks at 2800–3050 that was seen on the modified nanoparticles was related to C–H stretching vibration of grafted silane that was not exist in the pure nanoparticles^45–47^. Based on the results, grafting of silane molecules on the nanoparticles surface was confirmed.

**Figure 2:**
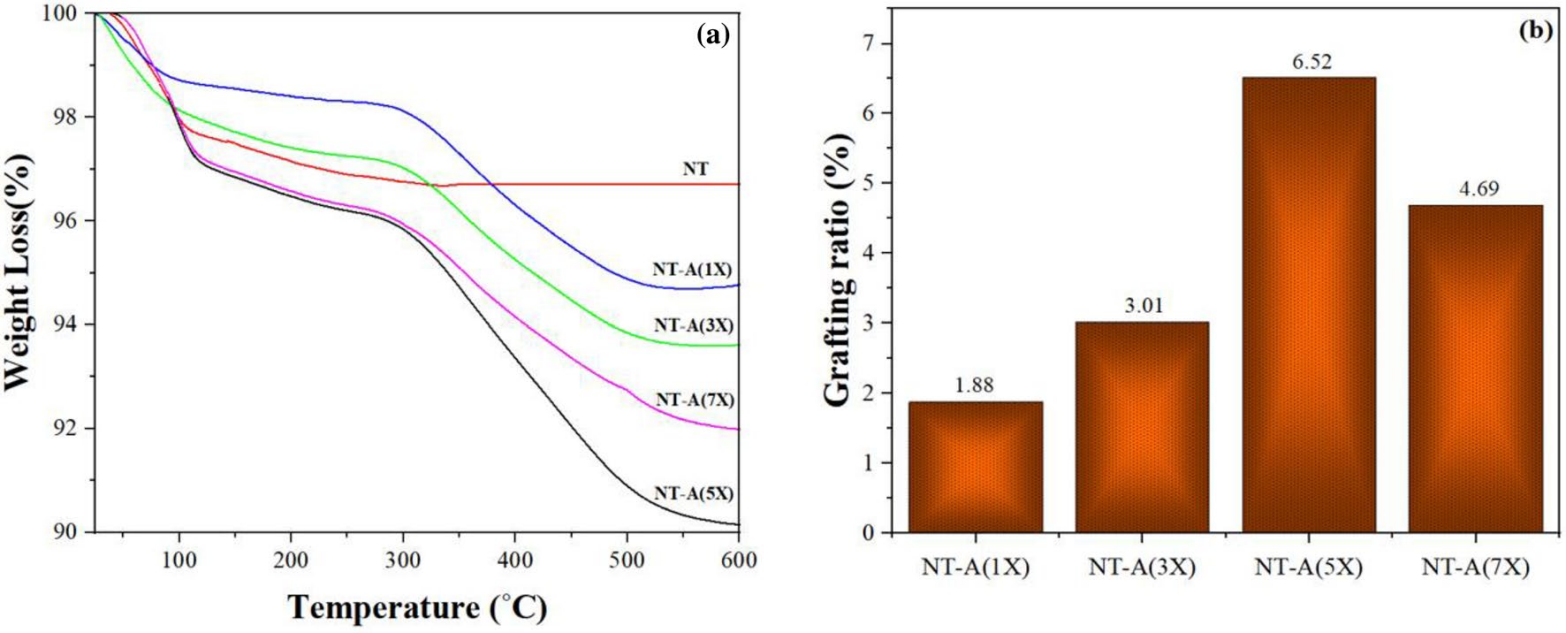
(**a**) TGA analysis and (**b**) grafting rates of pure TiO_2_ and modified TiO_2_ nanoparticles.

Figure 2 shows the TGA curves of the pure and surface modified nanoparticles (Samples prepared according to Eq. 1). Slight weight loss between 25 and 140 °C is likely because of water vaporization that physically adsorbed to the surface of TiO_2_ nanoparticles. This weight loss was approximately 2.46 and 3.08% for pure and silanized nanoparticles. The increment in the weight loss of the modified nanoparticles in this range was corresponded to the degradation of low molecular weight organic maters that absorbed during silanization process. The weight loss obtained at 140–335 °C was related to degradation of covalently bonded hydroxyl groups on TiO_2_ surface. The weight loss in this range remained constant after surface modification of nanoparticles while the OH content should be decreased due to their consumption during silane hydrolysis. This was attributed to placing of new/free OH groups on the surface that are related to the attachment of hydrolyzed silane molecules. In fact, these free OH groups have not contributed in condensation reactions.

It is evident that the pure nanoparticles does not show any weight loss at temperature range of 330–600 °C that is due to their highly stable inorganic structure that degrade at so much higher temperatures. However, the weight loss in this range for modified nanoparticles was corresponded to degradation of grafted and condensed silane molecules on the nano TiO_2_ surface that confirms successful silanization of BTESPA on the nanoparticles surface.

The highest silane grafting ratio was obtained at silane concentration of 5X. On this basis, the sample was selected as the best surface modified nano TiO_2_ and used for the next analysis and tests.

**Figure 3:**
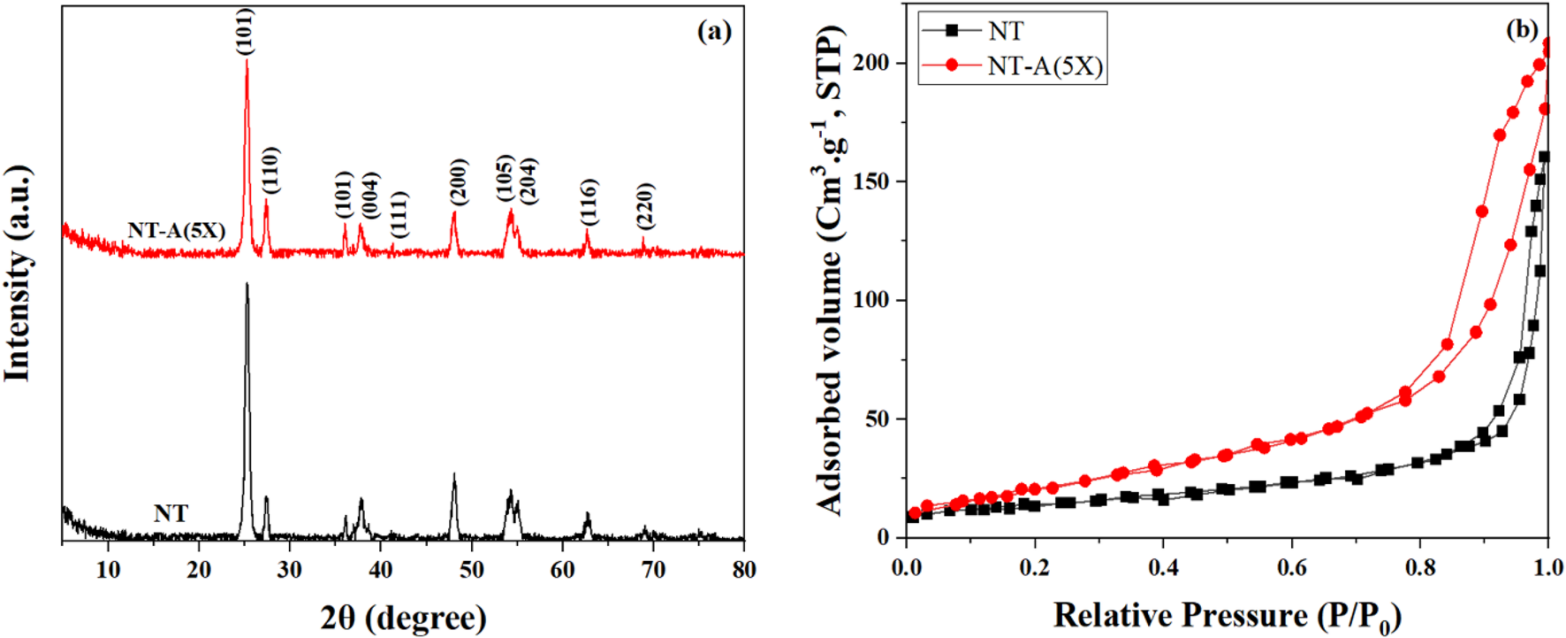
(**a**) XRD pattern and (**b**) N_2_ adsorption–desorption isotherms of NT and NT-A(5X) samples.

The XRD patterns of the pure and modified nanoparticles are shown in Fig. 3a. After silanization, the modified nanoparticle exhibited similar patterns to the pure TiO_2_ sample. As shown, both samples demonstrated the reflections for the anatase phase including: (101), (004), (200), (105), (204), (116), (220) planes placed at 25.3, 37.9, 48.0, 53.7, 54.8, 62.6, 68.7 and 70.2°, respectively^48^. The samples also depicted three small peaks placed at 27.4, 36.1 and 41.3° that were related to the rutile phase^49^. After stimulation of photocatalysts, the electron–hole pairs are formed but the electrons tend to come back to their holes. However, in presence of rutile phase, the holes migrate to this phase that this eliminates recombination of electron and holes (i.e. illustration of photocatalytic properties). It is obviously seen that the modification process did not affect the crystalline phase and size of nanoparticles (i.e. in the range of 14 − 16 nm).

Figure 3b and Table 2 show the BET results for the pure and modified nanoparticles. Both of the nanoparticles showed type IV Langmuir isotherm that confirms their mesoporous structure. The specific surface area of particles increased from 53.04 to 75.12 m^2^ /g (41.62% increment) by silane grafting. It was attributed to the steric stabilization of nanoparticles by the attached silane molecules that decreased nanoparticles agglomeration and particles size. In addition, the reduction in the pore diameter after surface modification was related to the filling of the pores by silane molecules^50^.

**Table 2:** The results of BET analysis.

Nano TiO_2_	Specific surface area (m^2^ /g)	Average pore diameter (nm)	Total pore volume (cm^3^ /g)
NT	53.04	25.61	0.243
NT-A(5X)	75.12	21.45	0.322

**Figure 4:**
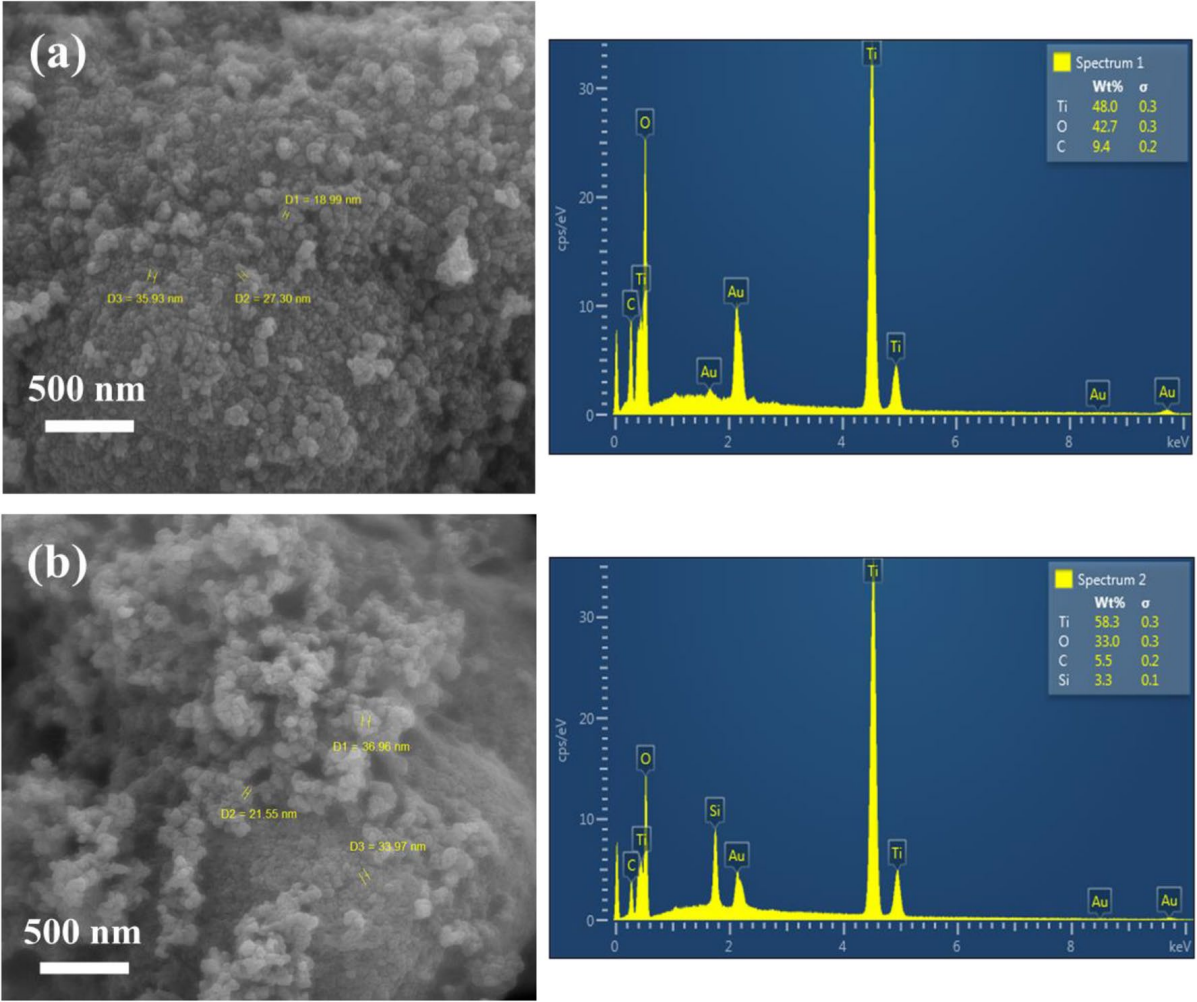
FESEM-EDX analysis of (**a**) NT and (**b**) NT-A(5X) samples.

**Figure 5:**
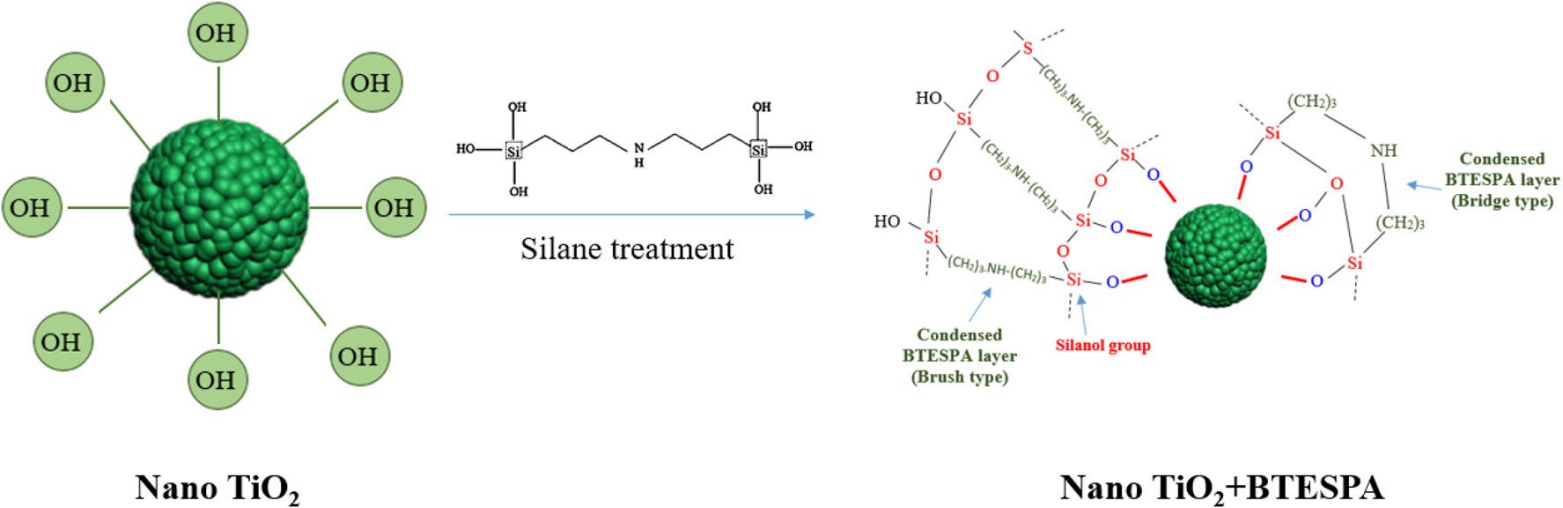
Schematic for reaction of hydrolyzed BTESPA and nano TiO_2_ and formation of condensed silane layer on nano TiO_2._

FE-SEM images of the pure and modified TiO_2_ photocatalysts are presented in Fig. 4a,b. The pure TiO_2_ contains aggregated nanoparticles but the size of single particles were lower before silane grafting. This was corresponded to the big molecular structure of BTESPA molecule. The elemental analysis (EDS) of the nanoparticles illustrated the presence of Si element in the modified TiO_2_ that confirmed successful grafting of silane molecules. Figure 5 shows a schematic for the silane grafting of the TiO_2_ nanoparticles.

### Characteristics of nanocomposites

**Figure 6:**
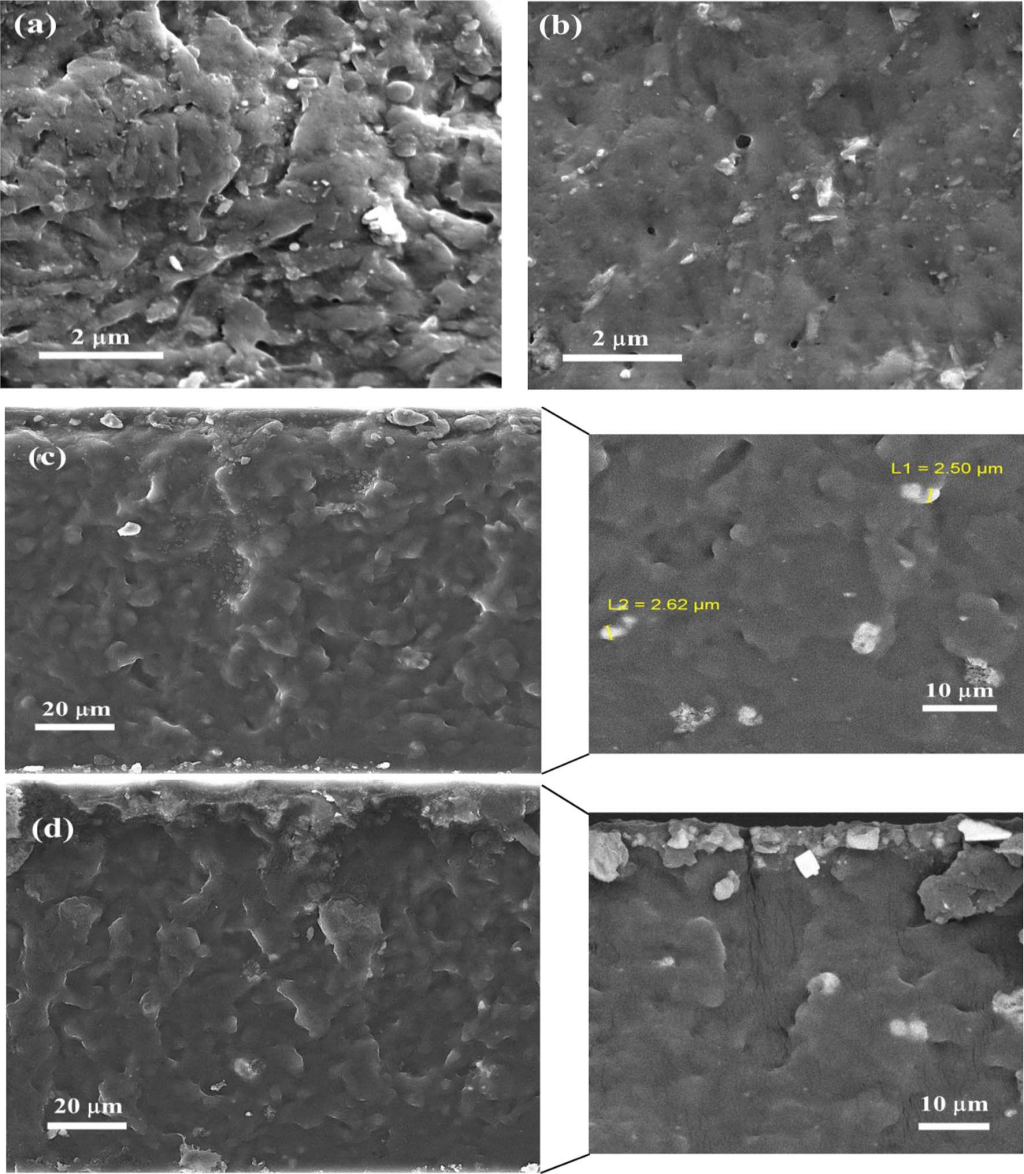
SEM analysis of nanocomposites; (**a**) AC-NT3, (**b**) AC-NT3-A(5X), (**c**) cross-sectional images of AC-NT3, (**d**) cross-sectional images of AC-NT3-A(5X) samples.

Figure 6a,b exhibit the surface images of AC-NT3 and AC-NT3-A(5X) samples. It is seen that the surface of the film contained pure TiO_2_ nanoparticles became clumped because of the agglomeration of the nanoparticles and incompatibility of the hydrophilic pure nanoparticles to the hydrophobic acrylic matrix. However, after modifying the nanoparticle with BTESPA, the surface of prepared film became considerably uniform.

Figure 6c,d display cross-sectional images of the fractured films. It is obvious that the river like lines increased in the modified sample. This was attributed to the improved interfacial interactions between acrylic matrix and silanized nanoparticles^18,32^. Moreover, in both samples surface immigrated particles were observed.

**Figure 7:**
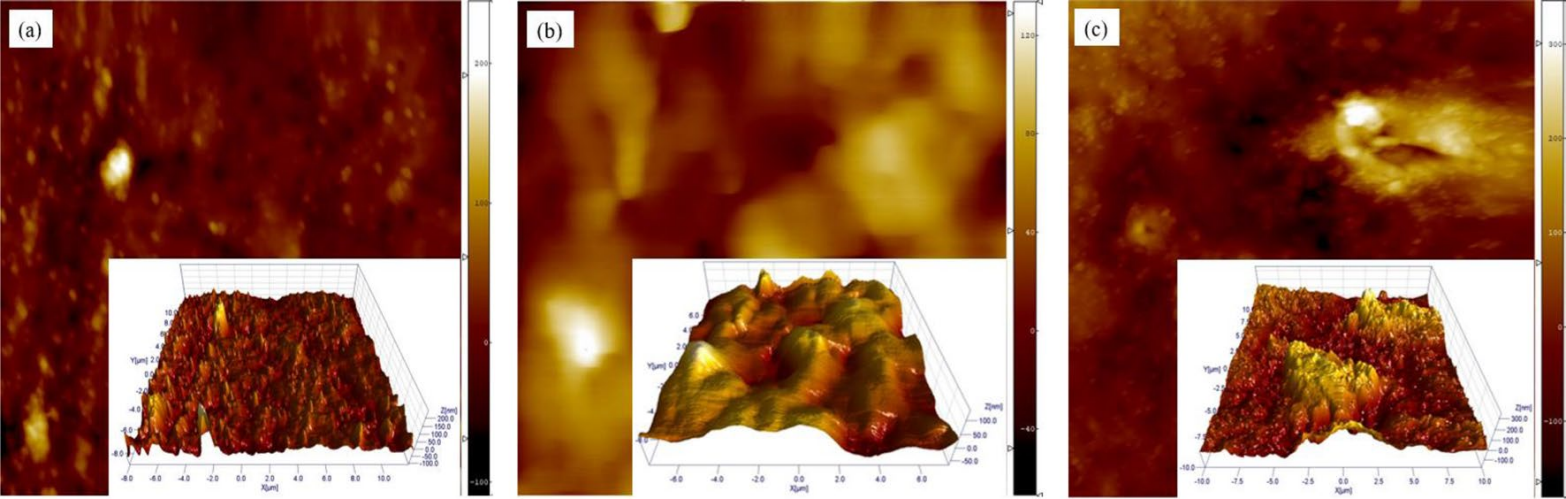
2D and 3D AFM images of (**a**) AC, (**b**) AC-NT3 and (**c**) AC-NT3-A(5X) films.

AFM analysis was used to analyze the surface roughness and morphology of the nano films. Figure 7 illustrates two-dimensional (2D) and three-dimensional (3D) surface morphologies of pure acrylic, pure TiO_2_ and silane-modified TiO_2_ nano films. Table 3 reports the exact quantities of roughness of the films obtained from the AFM analysis. The root mean square (RMS) and grain size of the roughness increased with addition of the nanoparticles to acrylic film. The surface roughness (*R*_*q*_) is a critical factor in the physical behaviors of thin films. The pure TiO_2_ containing nano film had a smooth surface morphology with a well-defined crystallinity. The surface roughness, as a key element in coating quality, was enhanced by addition of the modified nanoparticles to acrylic film, due to their better dispersion in the polymeric matrix. Additionally, appropriate nanoparticle dispersion demonstrates their more more substantial polymer/particle interactions that leads to improvement in the mechanical characteristics. Moreover, water molecules are more easily trapped and absorbed by the surface of nanoparticles by increasing in the surface roughness that this enhances the hydrophilicity of the modified nanoparticles. It also allows more contact with contaminants on the coating surface which could increase the photocatalytic degradation efficiency.

**Table 3:** Roughness parameters of acrylic films.

Samples	Roughness parameters (nm)			
	*R*_*a*_	*R*_*q*_	*R*_*sk*_	*R*_*ku*_
AC	13	18.4	0.588	4.06
AC-NT3	24.6	29.8	0.071	− 0.346
AC-NT3-A(5X)	66.1	84.5	0.924	0.46

### Thermal/mechanical properties of nanocomposites

**Figure 8:**
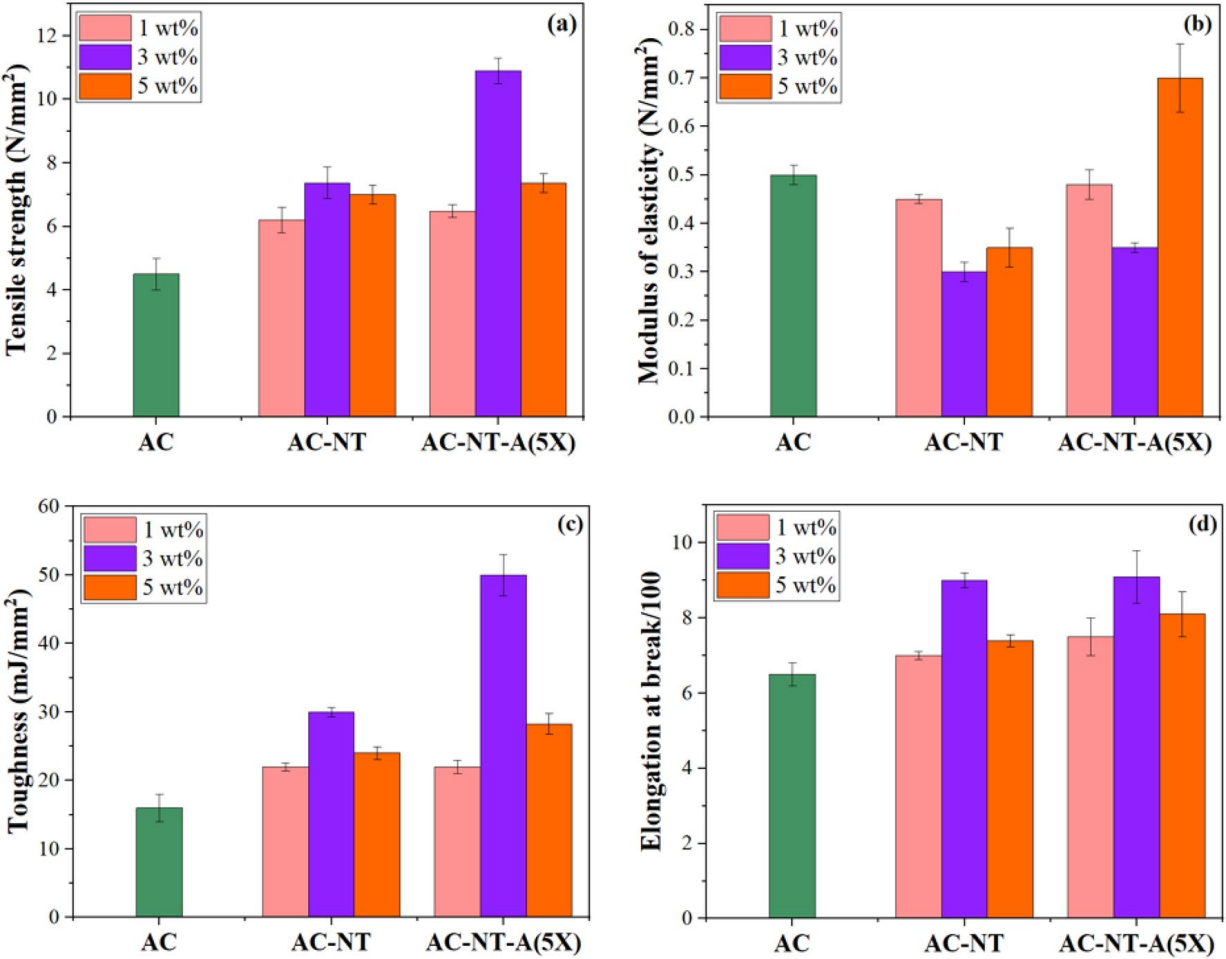
Tensile properties of pristine and nanoparticle contained acrylic films; (**a**) tensile strength, (**b**) modulus, (**c**) toughness and (**d**) elongation at break.

Figure 8 shows the flexural behavior of the pure and modified acrylic films at different nanoparticle contents. It is clearly seen that in presence of the pure and modified nanoparticles the tensile strength of the acrylic film increased (see Fig. 8a). This was attributed to the reinforcing effect of nanoparticles. However, the modified TiO_2_ nanoparticles improved it much more (up to 11 MPa) due to the improved interfacial interactions between polymer chains and silane grafted nanoparticles. The maximum flexural strength was obtained at filler loading content of 3 wt%. It was found that increasing in the pure nano TiO_2_ content had negligible effect on the flexural strength of the acrylic film. The modulus of acrylic film decreased by incorporation of the pure and modified nanoparticles except in the case of using 5 wt% of modified nanoparticles (see Fig. 8b). In the case of using 5 wt% of modified nanoparticles, some of them migrated to the surface (i.e. similar to self- stratification or self-layering of nanoparticles towards film surface) but most of the nanoparticles remained in the film and increased modulus due to improved interfacial interactions to acrylic chains. The flexural toughness and elongation at break of the nanoparticle containing acrylic films showed improvement compared to the pure acrylic film. The enhancement was significant at 3 wt% loading of the nanoparticles (see Fig. 8c,d).

**Figure 9:**
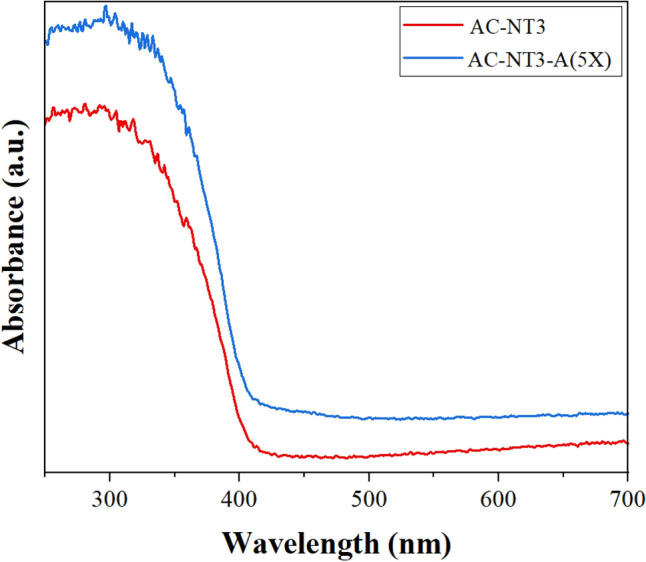
UV–Vis DRS spectra for AC-NT3 and AC-NT3-A(5X) nanocomposites.

The light absorption characteristics of the pure and modified nano TiO_2_ containing acrylic films were examined by DRS analysis. Figure 9 shows the absorbance spectra of the films. It is clearly seen that surface modification of TiO_2_ nanoparticles caused improvement in the light absorbance of acrylic films in both UV and visible regions. This finding could confirm migration of some nanoparticles to the film surface that improved light absorbance.

**Figure 10:**
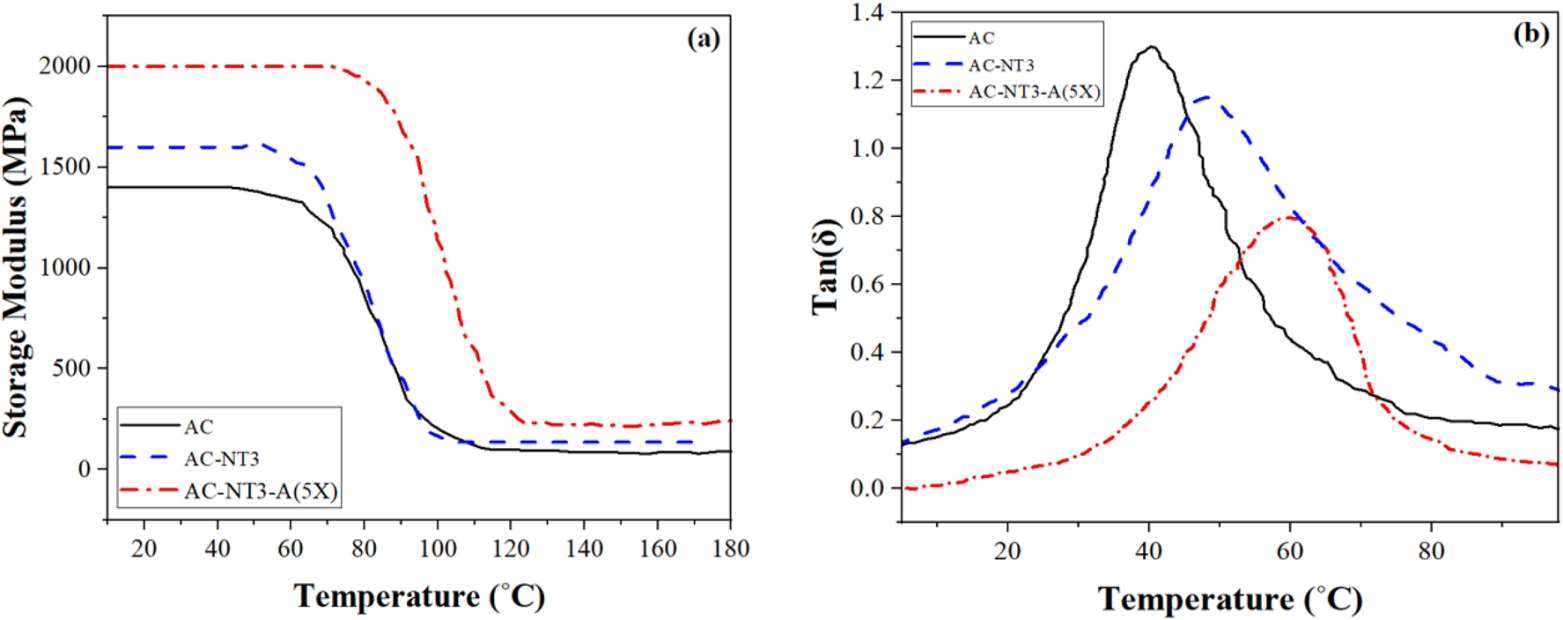
DMTA results; (**a**) storage modulus and (**b**) tan(δ) curves versus temperature for different acrylic films.

The DMTA results of the acrylic samples are presented in Fig. 10. It was observed that both of the nanoparticles improve storage modulus of the acrylic film, however, considerable increment was obtained in the case of using modified nano TiO_2_ (see Fig. 10a). This was directly corresponded to the improved interfacial interactions between polymer chains and silane grafted nanoparticles. A slight change in the T_g_ of the acrylic film (i.e. based on the turning point of the storage modulus curves) was achieved by inclusion of the pure nano silica while considerable enhancement in Tg was obtained by using modified nanoparticles in acrylic film. This was obvious in T_g_ results derived from tan(δ) curves (see Fig. 10b). Tan(δ) was decreased by incorporating nano fillers to the acrylic films. This was attributed to the interlocking between the nanoparticles and polymer chains that decreased movement of the chains under deformation. The decrement in the tan(δ) was much more in the modified nanocomposite due to enhanced interfacial interactions between nano fillers and polymeric matrix that limited chain movements.

**Figure 11:**
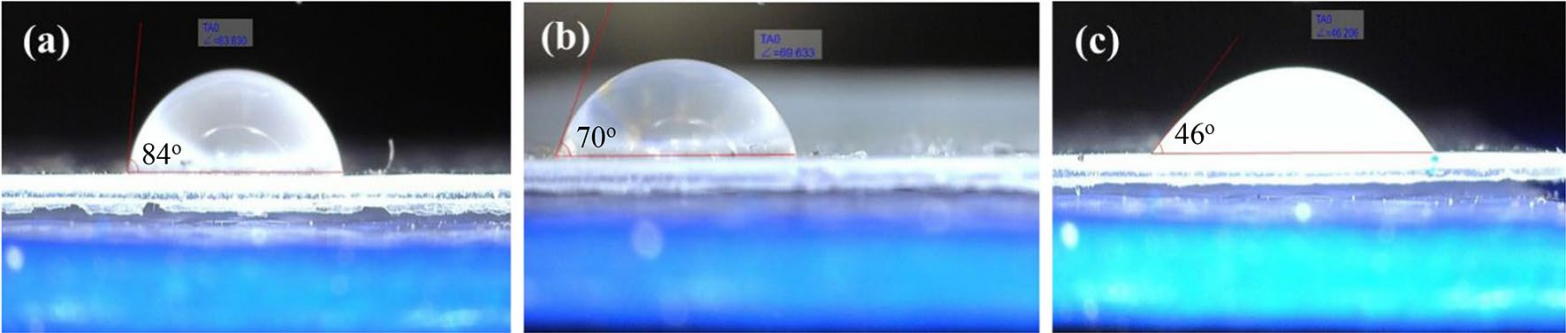
Contact angles of water droplets on the surface of; (**a**) pure AC, (**b**) AC-NT3, and (**c**) AC-NT3-A(5X).

Figure 11 shows the contact angle test results for the pristine and nanocomposite acrylic films. The inclusion of nanoparticles to acrylic film caused decrement in the water contact angle. In fact, the presence of pure hydrophilic nano TiO_2_ and probably its agglomeration in the acrylic film turned its nature to hydrophilic somehow. This finding was also in good correlation to the results of AFM analysis. The lowest contact angle was related to the modified nanocomposite film that showed more hydrophilicity. The change in this property could occur when the nanoparticles migrate to the surface of acrylic film to change its surface nature from relatively hydrophobic to hydrophilic. This was occurred due to presence of free hydrophilic NH and OH groups in BTESPA molecule (see Fig. 5 for better understanding). This claim was in line with previous researches that grafted nanoparticles by amino silanes^18,38^.

This finding supported the claim of self-stratification of some of the nanoparticles within acrylic film (i.e. FE-SEM results), because the pristine acrylic film has tendency to hydrophobic materials.

### Photocatalytic degradation of MB

**Figure 12:**
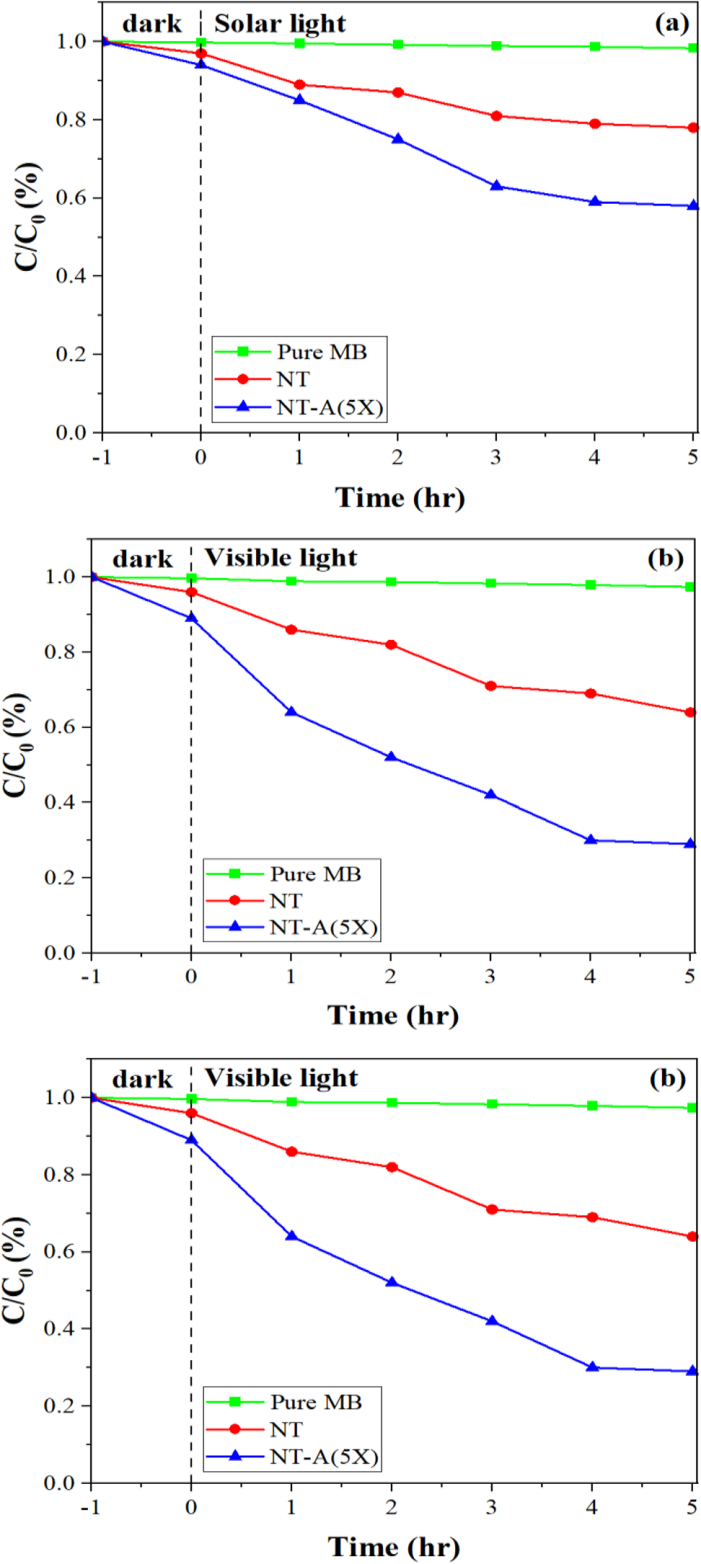
Photocatalytic degradation of MB in model wastewater in presence of TiO_2_ nanoparticles under (**a**) solar, (**b**) visible, and (**c**) UV lights.

The photocatalytic performances of the pure and modified TiO_2_ nanoparticles were determined by degradation of methylene blue in an aqueous solution under solar, visible and UV light sources. Figure 12 depicts the degradation contents of MB during 5 h. Under all light sources, the MB concentration experienced a slight decrease in absence of the nanoparticles because of the photolysis of MB molecules (i.e. 4.5, 2.5 and 1.98% under UV, visible and solar lights, respectively). In dark condition, the modified TiO_2_ nanoparticles demonstrated the highest capacity in adsorbing MB from the aqueous solution. According to the results, the maximum photocatalytic degradation of MB was obtained by modified TiO_2_ photocatalyst under all light sources while the minimum content was assigned to the pure TiO_2_ (see Fig. 12a–c)_._

**Figure 13:**
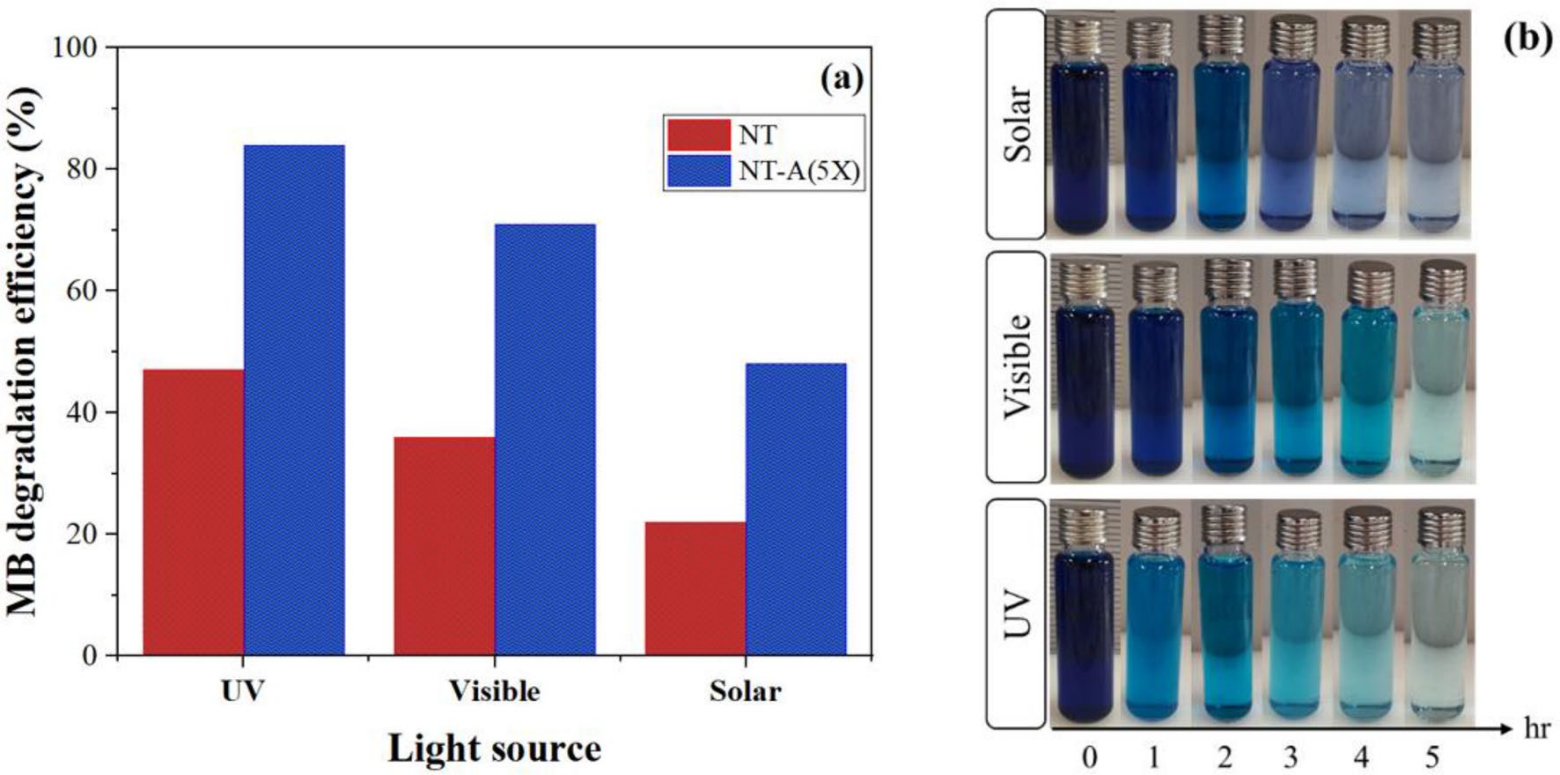
(**a**) The efficiency of photo-degradation of MB after five hours, (**b**) color change of MB aqueous solutions under different light sources throughout the photo-degradation process.

For better understanding, the results are shown in bar chart shape in Fig. 13a. The highest and lowest photocatalytic performances were obtained under UV and solar irradiation, respectively. Figure 13b shows the changes in blue color of MB solutions (i.e. pure MB solution and the solutions exposed to modified nano TiO_2_) through irradiation times.

**Figure 14:**
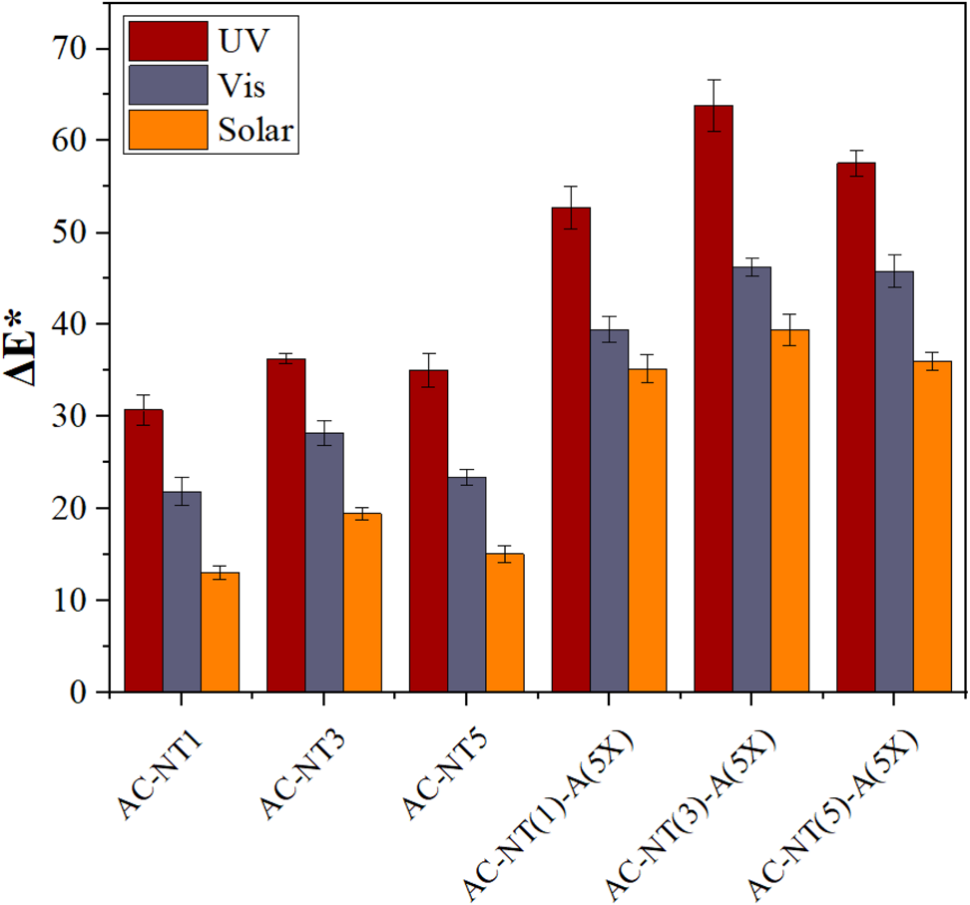
The color change (ΔE*) values of MB stains formed on different films under different light sources.

The photocatalytic performances of the acrylic films was evaluated by measurement of the color changes (ΔE*) of the methylene blue stains under UV irradiation. The results are shown in Fig. 14. It is evident that much more color changes (ΔE*) happened in the MB stains on acrylic films contained modified nanoparticles. It was also found that the highest color change (i.e. 65%) occurred in the film contained 3 wt% of silanized TiO_2_ nanoparticles. Besides, promising results for photocatalytic degradation of MB stains were obtained for nanocomposite films contained modified nanoparticles under visible and solar lights (i.e. of up to 47 and 40%, respectively).

The results also proves the claim of self-stratification of nanoparticles to the surface of the acrylic films that happened more in the case of silane grafting of the TiO_2_ nanoparticles because it tends to migration of some of the nanoparticles to the film surface. The results were in good correlation to the contact angle and AFM and FE-SEM results.

## Conclusions

In this work, TiO_2_ nanoparticles were surface modified by a bifunctional amine-based silane (BTESPA). The effects of grafting of a bifunctional amino silane on the nanoparticles and its influences on the surface properties and photocatalytic performances was evaluated. The pure and modified nanoparticles were applied at different concentrations to acrylic latex and the mechanical and photocatalytic performances of the prepared films were evaluated. The primary outcomes were concluded:

- The highest BTESPA silane grafting rate was obtained at silane concentration of 5X (about 6.52%).
- Silane grafted TiO_2_ nanoparticles caused 47% increment in the tensile strength of acrylic film compared to the pure nanoparticles.
- Modified nanocomposites (at filler loading content of 3 wt%) showed 18.4% increment in T_g_ and 10% decrement in tan (δ) compared to the nanocomposite contained pure TiO_2_.
- The maximum photocatalytic degradation efficiency of MB under solar light was related to the sample that contained amino silane grafted TiO_2_ under UV light (84%), whereas the minimum content was related to the sample contained pure TiO_2_ nanoparticles (22.11%).
- Silane grafting of TiO_2_ nanoparticles tends to more hydrophilic surface of the acrylic film. Water contact angles of 46.2, 83.83, and 69.63° was obtained for pure acrylic, acrylic film contained 3 wt% of pure nano TiO_2_ and modified nano TiO_2_, respectively.
- According to FESEM-EDX results, the existence of silicon, carbon, and nitrogen elements in the modified TiO_2_ with silane demonstrated a successful surface modification, as well as an increased aggregate size compared to the pure TiO_2_.
- The photocatalytic degradation behavior of the silane grafted TiO_2_ significantly improved compared to the pure TiO_2_ under UV, solar and visible lights. During a degradation time of 5 h, the photocatalysts demonstrated their maximum photocatalytic degradation efficiency under UV light (47 and 84% for TiO_2_ and modified TiO_2_, respectively). Under visible light, the photocatalytic degradation efficiency of modified nano TiO_2_ (71%) was higher than that of the pure nano TiO_2_ (36%). It was about 48 and 22% for the modified and pure TiO_2_ nanoparticles under solar light.

## Data Availability

All data generated or analysed during this study are included in this published article [and its supplementary information files].
